# Externalisierendes Problemverhalten mit Freund:innen im Jugendalter: Welche Rolle spielen Faktoren der emotionalen Unbeteiligtheit?

**DOI:** 10.1007/s00103-024-03850-4

**Published:** 2024-03-13

**Authors:** Sara-Marie Schön, Monika Daseking

**Affiliations:** Professur für Entwicklungspsychologie und Pädagogische Psychologie, Helmut-Schmidt-Universität/Universität der Bundeswehr Hamburg, Am Stadtrand 50, 22159 Hamburg, Deutschland

**Keywords:** CU Traits, Externalisierendes Problemverhalten, Jugendliche, Peer-Effekte, Affektive Psychopathie, CU traits, Externalizing problem behavior, Adolescents, Peer effects, Affective psychopathy

## Abstract

**Einleitung:**

Eine Erklärung dafür, dass Jugendliche externalisierendes Problemverhalten (regelverletzendes und dissoziales Verhalten) zeigen, sind affektive Beeinträchtigungen. Ein abgeflachter Affekt ist ein Kernmerkmal der Callous-Unemotional Traits (CU-Traits, dt.: emotionale Unbeteiligtheit). CU-Traits setzen sich aus den 3 Faktoren *Callousness* (Mangel an Empathie und Reue), *Uncaring *(gleichgültige Haltung ggü. der Erfüllung von Aufgaben und den Gefühlen anderer) und *Unemotional *(oberflächlicher oder abgeflachter Affekt) zusammen. Externalisierendes Problemverhalten findet im Jugendalter zumeist in Gruppen statt. Daher ist es wichtig, den Zusammenhang von CU-Traits und externalisierendem Problemverhalten zu betrachten, das explizit gemeinsam mit bzw. in Anwesenheit von Freund:innen gezeigt wird. Diese Perspektive fehlt bisher in der Forschung zu CU-Traits. Die vorliegende Studie hat das Ziel, diese Forschungslücke zu schließen.

**Methode:**

Von *N* = 169 Jugendlichen (54 % weiblich; *M* = 14,95 Jahre) wurden im Zeitraum von Juni 2021 bis März 2023 Selbstberichtsdaten mit einer online oder handschriftlich ausfüllbaren Fragebogenbatterie erhoben, die neben CU-Traits (ICU) auch Einschätzungen zu regelbrechendem und dissozialem Verhalten mit Freund:innen (CBCL: YSR 11–18 R) enthielten.

**Ergebnisse:**

Ausschließlich die Subskala *Callousness* trägt zur Erklärung von externalisierendem Problemverhalten mit Freund:innen bei (6 % Varianzaufklärung).

**Diskussion:**

Die 3 Faktoren der CU-Traits sollten getrennt voneinander betrachtet werden. Außerdem scheint es bei der Untersuchung von externalisierendem Problemverhalten wichtig zu sein, ob es allein oder gemeinsam mit bzw. in Anwesenheit von Freund:innen gezeigt wird. Limitationen der Studie, Ideen für weiterführende Forschung und praktische Implikationen werden diskutiert.

## Abstract (en)

**Introduction:**

Affective impairments/disorders are one explanation as to why adolescents exhibit externalizing problem behavior (rule-breaking and dissocial behavior). Impaired affect is a core feature of callous-unemotional traits (CU traits). CU traits are composed of three factors: *Callousness* (lack of empathy and remorse), *Uncaring* (indifferent attitude toward task performance and the feelings of others), and *Unemotional* (superficial or flattened affect). Externalizing problem behaviors mostly occur in groups during adolescence. Therefore, it is important to consider the relationship between CU traits and externalizing problem behaviors that are exhibited explicitly in collaboration with or in the presence of friends. This perspective has been lacking in research on CU traits. The present study aims to fill this research gap.

**Methods:**

Self-report data were collected between June 2021 and March 2023 from *N* = 169 adolescents (54% female; *M* = 14.95 years) using a set of questionnaires that could be completed online or as a paper-pencil version. In addition to CU traits (ICU) the set of questionnaires included assessments of rule-breaking and dissocial behavior with friends (CBCL: YSR 11–18 R).

**Results:**

Only the subscale *Callousness* contributes to the explanation of externalizing problem behaviors with friends (6% variance explained).

**Discussion:**

The results indicate that the three factors of CU-traits should be considered separately. In addition, when examining externalizing problem behavior, one should consider whether it is exhibited alone or together with or in the presence of friends. Limitations of the study, ideas for further research, and practical implications are discussed.

## Einleitung

Externalisierende Problemverhaltensweisen sind Kernmerkmale der Störung des Sozialverhaltens und hyperkinetischer Störungen. Sie sind gekennzeichnet durch destruktive, nach außen gerichtete Handlungen wie regelverletzendes, dissoziales, aggressives und straffälliges Verhalten sowie Aufmerksamkeitsstörungen, Impulsivität, Überaktivität und emotionale Instabilität. Die Arten von externalisierendem Problemverhalten, auf denen der Fokus in der vorliegenden Arbeit liegt und die im Folgenden unter *externalisierendem Problemverhalten* zusammengefasst werden, sind (a) regelverletzendes und (b) dissoziales Verhalten. Regelverletzendes Verhalten beinhaltet Substanzmissbrauch, sexualisiertes Verhalten, Stehlen, Lügen, Zerstörung und Kontakt zu älteren/regelverletzenden Jugendlichen [1]. Dissoziales Verhalten weist Überschneidungen mit regelverletzendem Verhalten auf, beinhaltet zusätzlich jedoch schwereres regelverletzendes Verhalten in sozialen Interaktionen (z. B. Bedrohung und körperliche Angriffe; [1]). Beides ist bei den meisten Jugendlichen mit negativen moralischen Emotionen (z. B. Schuld und Scham) assoziiert, da es in Kontrast zu ihren moralischen Standards steht [2]. Zur Vermeidung negativer moralischer Emotionen wird das eigene Handeln reguliert, wodurch weiteres externalisierendes Problemverhalten in vielen Fällen verhindert wird. Die deutsche Kriminalstatistik zeigt jedoch, dass im Jahr 2022 [3] 189.149 Jugendliche im Alter zwischen 14 und 18 Jahren als straffällig beziffert worden sind. Eine mögliche Erklärung dafür könnte in der psychischen Konstitution dieser Jugendlichen liegen: So könnten affektive Beeinträchtigungen dazu führen, dass keine oder nur eine unzureichende Handlungsregulation erfolgt. Abgeflachte prosoziale Emotionalität ist ein Kernmerkmal der sogenannten Callous-Unemotional Traits (CU-Traits, dt.: emotionale Unbeteiligtheit) und kann als Zusatzkodierung zur Diagnose „Störung des Sozialverhaltens“ ausgewählt werden [4, 5].

### Callous-Unemotional Traits

Das Konstrukt der CU-Traits entspricht der affektiven Dimension von Psychopathie im Erwachsenenalter [6]. CU-Traits sind unter anderem mit einem Mangel an Schuldgefühlen, Reue und Sorge um die Gefühle anderer assoziiert [7]. Zudem weisen Jugendliche mit hohen Ausprägungen von CU-Traits normabweichende emotionale und kognitive Reaktionen zum Beispiel auf Bestrafung und Stresssignale bei anderen auf [8]. Dies führt dazu, dass CU-Traits maßgeblich zur Entwicklung und Aufrechterhaltung von – vor allem – besonders schwerem externalisierenden Problemverhalten, wie reaktiver Aggression, Bullying, Cyberbullying und Straffälligkeit, beitragen [9–12].

Als gängiges Instrument zur Messung von CU-Traits im Kindes- und Jugendalter gilt das Inventory of Callous-Unemotional Traits (ICU; [9, 10, 13–15]). Das ICU misst CU-Traits auf 3 Subskalen, die folgende inhaltliche Faktoren abbilden:

- *Callousness *(Mangel an Empathie und Reue),
- *Uncaring* (gleichgültige Haltung ggü. Erfüllung von Aufgaben und Gefühlen anderer) und
- *Unemotional* (oberflächlicher oder abgeflachter Affekt).

Die Werte der 3 Subskalen können zu einem Gesamtscore zusammengefasst werden [7]. Bisherige Forschungsergebnisse zeigen, dass sich die Zusammenhänge der 3 Faktoren mit externalisierendem Problemverhalten unterscheiden [16]. Anders als die Faktoren *Callousness *und *Uncaring* zeigt der Faktor *Unemotional* keine konsistenten Assoziationen mit Verhaltens- und schulischen Anpassungsproblemen [9], jedoch mit Beeinträchtigungen in allgemeinen emotionalen Kompetenzen [10].

### Externalisierendes Problemverhalten mit Freund:innen

Jugendkriminalität ist ein Gruppenphänomen [17, 18] und, dass der Kontakt mit Freund:innen, die externalisierende Verhaltensprobleme haben, mit vermehrtem externalisierenden Problemverhalten assoziiert ist, konnte bereits mehrfach gezeigt werden [19–22]. Erklärungen für diesen negativen Einfluss der Freund:innen bieten z. B. die soziale Lerntheorie [23] und die Theorie der sozialen Identität [24].

Im Rahmen der sozialen Lerntheorie wird angenommen, dass Freund:innen, die externalisierendes Problemverhalten zeigen, als Vorbilder dienen, wodurch externalisierendes Problemverhalten gerechtfertigt und sogar als wünschenswert definiert wird [23]. Externalisierendes Problemverhalten wird zusätzlich dadurch gefördert, dass es innerhalb des Freundeskreises belohnt und nicht sanktioniert wird. Die Belohnung zeigt sich zum Beispiel durch anerkennende Gesten oder Lachen, was als positive Verstärkung wirkt [19].

Die Theorie der sozialen Identität [24] unterscheidet zwischen persönlicher Identität und sozialer Identität, wobei Letztere auf Kategorisierungen einer sozialen Gruppe (z. B. regelbrechend) basiert. Gruppenkonformes Verhalten wird nicht als Resultat von sozialem Druck gesehen, sondern resultierend aus der Integration von Teilen der sozialen Identität in die persönliche Identität. Wenn es also zur Identität des Freundeskreises gehört, Regeln zu brechen, und dies innerhalb der Gruppe als wünschenswert definiert wird, integrieren die Gruppenmitglieder diese Definition in ihre persönliche Identität und verhalten sich entsprechend, indem sie Regeln brechen.

Die soziale Lerntheorie als auch die Theorie der sozialen Identität zeigen exemplarisch, dass externalisierendes Problemverhalten, das gemeinsam bzw. im Beisein von Freund:innen gezeigt wird, auf anderen, komplexeren Mechanismen beruht als externalisierendes Problemverhalten, das alleine gezeigt wird. Daher ist es wichtig, bei der Messung von externalisierendem Problemverhalten zu berücksichtigen, ob dies allein oder in Anwesenheit von Freund:innen gezeigt wird. Diese Perspektive fehlt jedoch in der Forschung zu CU-Traits bisher meist. Daher liegt das Ziel der vorliegenden Studie auch darin, dazu beizutragen, diese Forschungslücke zu schließen. Aufbauend auf den Ergebnissen, die zeigen, dass sich die 3 Faktoren der CU-Traits in ihren Zusammenhängen unterscheiden [9, 10], soll zudem geprüft werden, ob es für die Erklärung von externalisierendem Problemverhalten mit Freund:innen relevant ist, die Faktoren getrennt zu betrachten.

Daraus ergeben sich folgende Forschungsfragen: Erklären CU-Traits Varianz in externalisierendem Problemverhalten, das gemeinsam mit Freund:innen begangen wird, und tragen die 3 Faktoren der CU-Traits in unterschiedlichem Ausmaß dazu bei?

Die folgenden Annahmen wurden aus den Forschungsfragen vor dem Hintergrund des explorativen Ansatzes der vorliegenden Arbeit abgeleitet. Grundsätzlich ist davon auszugehen, dass CU-Traits auch mit externalisierendem Problemverhalten zusammenhängen, wenn es gemeinsam mit Freund:innen gezeigt wird. Da Jugendliche mit hohen Ausprägungen von CU-Traits häufiger Freund:innen mit externalisierenden Verhaltensproblemen haben als Jugendliche mit niedrigen Ausprägungen von CU-Traits [25], ist der Zusammenhang möglicherweise noch stärker ausgeprägt als für externalisierendes Problemverhalten, das alleine begangen wird. Auf der anderen Seite ist es möglich, dass die im Rahmen der sozialen Lerntheorie [23] und der Theorie der sozialen Identität [24] beschriebenen Mechanismen, die externalisierendes Problemverhalten mit Freund:innen erklären, aufgrund höherer Ausprägungen von CU-Traits weniger wirksam sind (z. B. geringere Empfänglichkeit für emotionale Hinweisreize aus der Gruppe). Hierbei ist die separate Betrachtung der 3 Faktoren von besonderer Bedeutung. Der Mangel an Empathie, durch den der Faktor *Callousness *unter anderem gekennzeichnet ist [7], kann dazu führen, dass die Konsequenzen für Opfer von externalisierendem Problemverhalten weniger oder sogar gar nicht beachtet werden. Er kann aber möglicherweise auch dazu führen, dass die Emotionen, Gedanken und Motive der Mitglieder einer sozialen Gruppe weniger oder gar nicht wahrgenommen werden und ihr Einfluss auf das individuelle Verhalten dadurch schwächer ist. Ähnliches trifft eventuell auf den Faktor *Uncaring* zu, der unter anderem mit einer gleichgültigen Haltung gegenüber den Gefühlen anderer und der Erfüllung von Aufgaben assoziiert ist [7]. Durch die Gleichgültigkeit wirken sich die Erwartungen der sozialen Gruppe möglicherweise weniger oder sogar gar nicht auf das individuelle Verhalten aus. Für den Faktor *Unemotional *ist der schwächste Zusammenhang zu erwarten, zum einen aufgrund bisheriger Ergebnisse, die zeigen, dass er nicht konsistent mit Verhaltensproblemen assoziiert ist [9, 10], und zum anderen, da der Faktor eine schwache externe Validität aufweist [16].

## Methoden

### Teilnehmende und Vorgehen

Für die Datenerhebung (Juni 2021 bis März 2023) wurden Schulen unterschiedlicher Schulformen in Deutschland (Hamburg und Nordrhein-Westfalen) kontaktiert, wobei sich die Rekrutierung – vermutlich aufgrund der Coronapandemie – relativ schwierig gestaltet hat und daraus Einschränkungen hinsichtlich der Heterogenität der Stichprobe entstanden sind. Zusätzlich wurden im Juni und Juli 2021 sowie im März 2023 Daten eines in Deutschland und in der Schweiz durchgeführten Evaluationsprojektes verwendet. Zwischen den in Deutschland und in der Schweiz rekrutierten Jugendlichen sowie zwischen den in Schulen und im Rahmen des Evaluationsprojekts erfassten Daten lagen keine systematischen Unterschiede vor, weshalb die Daten für die hier vorgenommenen Analysen zusammengefasst wurden.

Es wurde eine Fragebogenbatterie verwendet, die online oder handschriftlich von den Jugendlichen ausgefüllt wurde. Insgesamt sollten ursprünglich *N* = 323 Jugendliche an der Studie teilnehmen, von diesen lehnten *n* = 2 die Teilnahme aktiv ab und *n* = 154 mussten ausgeschlossen werden, da sie die Bearbeitung vorzeitig abgebrochen hatten (*n* = 133) oder nicht in den definierten Altersbereich passten (*n* = 21). Es wurde geprüft, ob Jugendliche mit bestimmten soziodemografischen Merkmalen systematisch abgebrochen haben/ausgeschlossen wurden. Der Ein‑/Ausschluss hing insofern ausschließlich mit dem Geschlecht zusammen (χ^2^(1) = 7,78; *p* = 0,005), dass mehr Jungen aus- als eingeschlossen und mehr Mädchen ein- als ausgeschlossen wurden. Da es sich dabei um einen kleinen Effekt handelt (*V* = 0,18; *p* = 0,005; [26]), wird davon ausgegangen, dass die Ergebnisse dadurch nicht verzerrt wurden. Die relativ hohe Abbruchquote lässt sich dadurch erklären, dass die Datenerhebung in mindestens 2 Klassen von den Lehrerkräften aufgrund von Zeitmangel abgebrochen wurde. Über diesen systematischen Dropout hinaus fehlende Werte wurden durch mehrfache Imputation (vollständig bedingte Spezifikation) ersetzt. Die Parameterschätzungen wurden für die Punktschätzung gemittelt/gepoolt (kombinierter Wert).

Die finale Stichprobe umfasste *N* = 169 Jugendliche (54 % weiblich) mit einem Durchschnittsalter von 14,95 Jahren (*SD* = 1,7; Min. = 12 bis Max. = 18 Jahre). Etwas mehr als die Hälfte der Jugendlichen gab an, ein Gymnasium zu besuchen (*n* = 95), gefolgt von der Realschule (*n* = 41), der Stadtteilschule (*n* = 18), der Gesamtschule (*n* = 13) und der Berufsschule (*n* = 2). Die meisten der Jugendlichen wurden in Deutschland geboren (*n* = 160), gefolgt von Syrien (*n* = 4) und unter anderem Indien, Irak und Thailand (jeweils *n* = 1). Zudem gab ein Großteil der Jugendlichen an, zu Hause hauptsächlich Deutsch zu sprechen (*n* = 121), gefolgt von Russisch (*n* = 7), Türkisch (*n* = 5), Englisch (*n* = 4), Arabisch (*n* = 4) und Spanisch/Katalanisch (*n* = 2). Jeweils einmal genannt wurden z. B. Bosnisch, Französisch und Urdu. Jugendliche, die älter als 16 Jahre waren, gaben ihre informierte Zustimmung zur Teilnahme selbst. Bei Jugendlichen unter 16 Jahren wurde die Erlaubnis der Erziehungsberechtigten eingeholt. Die Teilnahme war freiwillig und konnte ohne Nachteile abgelehnt oder abgebrochen werden.

### Instrumente

#### Callous-Unemotional Traits

CU-Traits wurden mit der deutschen Übersetzung des Inventory of Callous-Unemotional Traits – Adolescents (ICU; [13, 15]) gemessen. Das ICU besteht aus 24 Items und misst CU-Traits auf den 3 vorab beschriebenen Subskalen *Callousness* (Bsp.-Item: „Die Gefühle anderer sind mir unwichtig“), *Uncaring* (Bsp.-Item: „Ich versuche, die Gefühle anderer nicht zu verletzen“) und *Unemotional *(Bsp.-Item: „Ich verstecke meine Gefühle vor anderen“). Obwohl die Faktorstruktur des ICU noch nicht abschließend geklärt zu sein scheint, deuten bisherige Ergebnisse für das Jugendalter auf ein Modell mit einem übergeordneten allgemeinen Faktor und den 3 Subfaktoren [10, 13, 27] hin. Die Validität und Reliabilität des ICU wurden bereits in mehreren Studien für das Kindes- und Jugendalter geprüft. In ihrer Metaanalyse berichten Cardinale und Marsh [16] von akzeptabler externer Validität und Reliabilität für den Gesamtscore sowie für die Subskalen *Callousness *und *Uncaring*. Cut-off-Werte sind für das Jugendalter bisher noch nicht etabliert. Erste Ergebnisse dazu zeigen jedoch, dass die Cut-off-Werte insbesondere bei bereits straffällig gewordenen Jugendlichen mit fortlaufender Delinquenz, Aggression und (wiederholter) Inhaftierung zusammenhängen [28]. Die Antworten werden auf einer 4‑stufigen Likert-Skala gegeben (0 = *trifft voll und ganz zu* bis 3 = *trifft überhaupt nicht zu*). Positiv formulierte Items wurden umcodiert, sodass höhere Werte einer höheren Ausprägung der CU-Traits entsprechen. Die interne Konsistenz in der vorliegenden Stichprobe betrug α = 0,75 für die Skala *Callousness*, α = 0,79 für die Skala *Uncaring* und α = 0,71 für die Skala *Unemotional.*

#### Externalisierendes Problemverhalten mit Freund:innen

Externalisierendes Problemverhalten, das gemeinsam mit Freund:innen begangen wird, wurde mit 13 Items auf Basis des „Youth Self-Report 11–18 R“ (YSR 11–18 R) der „Child Behavior Checklist“ (CBCL; [1]) gemessen. Bei allen Items der Skalen *Regelverletzendes Verhalten* und *Dissoziales Verhalten* wurde der Zusatz „Wenn du mit deinen Freund:innen zusammen bist …“ ergänzt (Bsp.-Item: „Wenn du mit deinen Freund:innen zusammen bist, zerstört ihr Dinge, die anderen gehören“). Die Skala *Regelverletzendes Verhalten* erfasst unter anderem Substanzmissbrauch und hat Überschneidungen mit der DSM-orientierten Skala *Dissoziales Verhalten* (z. B. gesetzesrelevante Regelverletzungen wie Diebstahl oder Sachbeschädigung und moralische Regelverletzungen wie Lügen). Die Antworten wurden auf einer 3‑Punkte-Likert-Skala gegeben (1 = *trifft nicht zu* bis 3 = *trifft genau oder häufig zu*). Höhere Werte entsprechen häufigerem externalisierenden Problemverhalten mit Freund:innen. Die Ergebnisse beider Skalen wurden addiert und ergaben für die vorliegende Stichprobe eine interne Konsistenz von α = 0,91.

#### Datenanalyse

Die statistischen Analysen wurden mit IBM SPSS Statistics (Version 27.0) durchgeführt. Zunächst wurden deskriptive und korrelative Analysen (Produkt-Moment-Korrelation nach Pearson, Testwert: *r*) gerechnet. Anschließend wurde geprüft, ob sich die Fisher-z-standardisierten-Korrelationen (Testwert: *z*) zwischen den Subskalen des ICU und externalisierendem Problemverhalten mit Freund:innen signifikant unterscheiden. Im zweiten Schritt wurden die Voraussetzungen für eine hierarchische lineare Regressionsanalyse (Bestimmtheitsmaß für die Anpassungsgüte der Regression: *R*^2^, Testwert der Varianzanalyse: *F*) geprüft, die anschließend für die Beantwortung der Fragestellung durchgeführt wurde.

## Ergebnisse

Die Ergebnisse der deskriptiven und korrelativen Analysen der Studienvariablen sind in Tab. 1 und Tab. 2 dargestellt. Die korrelative Analyse zeigte, dass die Subskalen *Callousness* (*r* = 0,25, *p* = 0,002) und *Uncaring *(*r* = 0,21, *p* = 0,008) positiv mit externalisierendem Problemverhalten mit Freund:innen zusammenhängen, die Subskala *Unemotional* jedoch nicht (*r* = 0,00, *p* = 0,979). Die Korrelationen der Subskalen *Callousness* sowie *Uncaring *mit externalisierendem Problemverhalten mit Freund:innen unterschieden sich nicht signifikant (*z* = 0,39, *p* = 0,700) und es handelte sich jeweils um schwache Effekte [26].

**Table 1:** 

Variablen (Anzahl Items)	Originaldaten					Kombinierter Wert mit imputierten Daten				
	*M*	*SD*	*SE*	Min.	Max.	*M*	*SD*	*SE*	Min.	Max.
Callousness (11)	9,9 (*N* = 161)	5,3	0,42	2	30	10,02	5,3	0,41	2	30
Uncaring (8)	8,5 (*N* = 168)	4,5	0,34	0	24	8,6	4,4	0,34	0	24
Unemotional (5)	8,1 (*N* = 161)	3,3	0,26	0	15	8,1	3,3	0,25	0	15
Externalisierendes Problemverhalten mit Freund:innen (13)	18,2 (*N* = 121)	6,3	0,58	13	39	18,6	5,6	0,43	13	39

Hohe Werte auf den Skalen *Callousness, Uncaring *und *Unemotional* (0 = *trifft voll und ganz zu* bis 3 = *trifft überhaupt nicht zu*) entsprechen hohen Ausprägungen von Callous-Unemotional-Traits. Hohe Werte in externalisierendem Problemverhalten mit Freund:innen (1 = *trifft nicht zu* bis 3 = *trifft genau oder häufig zu*) entsprechen häufigem externalisierenden Problemverhalten, das gemeinsam mit Freund:innen gezeigt wird

*M* Mittelwert, *SD* Standardabweichung, *SE* Standardfehler des Mittelwerts

**Table 2:** 

	(1)	(2)	(3)	(4)
(1) Callousness	1	–	–	–
(2) Uncaring	*r* = 0,34 (*p* < 0,001)	1	–	–
(3) Unemotional	*r* = 0,22 (*p* = 0,005)	*r* = 0,21 (*p* = 0,006)	1	–
(4) Externalisierendes Problemverhalten mit Freund:innen	*r* = 0,25 (*p* = 0,002)	*r* = 0,21 (*p* = 0,008)	*r* = 0,00 (*p* = 0,979)	1

Hohe Werte auf den Skalen *Callousness, Uncaring *und *Unemotional* (0 = *trifft voll und ganz zu* bis 3 = *trifft überhaupt nicht zu*) entsprechen hohen Ausprägungen von CU-Traits. Hohe Werte in externalisierendem Problemverhalten mit Freund:innen (1 = *trifft nicht zu* bis 3 = *trifft genau oder häufig zu*) entsprechen häufigem externalisierenden Problemverhalten, das gemeinsam mit/in Anwesenheit von Freund:innen gezeigt wird

*r* Pearsons Korrelationskoeffizient, *p* Signifikanzwert

Die Ergebnisse der Regressionsanalyse (Tab. 3) zeigten, dass ausschließlich die Subskala *Callousness* signifikant zur Aufklärung von Varianz in externalisierendem Problemverhalten mit Freund:innen beitrug (*R*^2^ = 0,06; *p* = 0,002; Änderung in *F* = 11,34; *p* < 0,001), die Subskalen *Uncaring* (Änderung in *F* = 3,11; *p* = 0,084) und *Unemotional *(Änderung in *F* = 0,96; *p* = 0,331) nicht. Mit einer Varianzaufklärung von 6 % handelt es sich um einen schwachen Effekt [26].

**Table 3:** 

	Schritt 1				Schritt 2				Schritt 3			
	*B*	*SE(B)*	β	*p*	*B*	*SE(B)*	β	*p*	*B*	*SE(B)*	β	*p*
Callousness	0,27	0,09	0,251	0,002	0,220	0,090	0,201	0,015	0,233	0,091	0,216	0,011
Uncaring	–	–	–	–	0,176	0,102	0,142	0,083	0,190	0,103	0,150	0,063
Unemotional	–	–	–	–	–	–	–	–	−0,130	0,134	−0,075	0,331
Änderung in *F*	11,34	–	–	< 0,001	3,11	–	–	0,084	0,96	–	–	0,331
*R*^2^	0,06	–	–	0,002	0,08	–	–	0,002	0,09	–	–	0,003

*B* Regressionskoeffizient B*, SE(B)* Standardfehler des Regressionskoeffizienten B, *β* standardisierter Koeffizient Beta*, F* Testwert der Varianzanalyse*, p* Signifikanzwert, *R*^2^ Bestimmtheitsmaß für die Anpassungsgüte der Regression

## Diskussion

Das Ziel der vorliegenden Studie war es zu prüfen, ob Callous-Unemotional Traits (CU-Traits) Varianz in externalisierendem Problemverhalten, das explizit gemeinsam mit Freund:innen gezeigt wird, erklären können und ob sich das Ausmaß der Varianzaufklärung zwischen den 3 Faktoren – *Callousness, Uncaring* und *Unemotional* – unterscheidet.

Es zeigt sich, dass ausschließlich der Faktor *Callousness*, also ein Mangel an Empathie und Reue, zur Aufklärung der Varianz in externalisierendem Problemverhalten mit Freund:innen beiträgt. Im Gegensatz dazu tragen eine gleichgültige Haltung gegenüber der Erfüllung von Aufgaben sowie den Gefühlen anderer (*Uncaring*) und ein oberflächlicher oder abgeflachter Affekt (*Unemotional*) nicht dazu bei, signifikant Varianz im externalisierenden Problemverhalten mit Freund:innen aufzuklären.

Damit stimmen die Ergebnisse der vorliegenden Studie mit bisherigen Ergebnissen [9, 10] überein, die zeigen konnten, dass die 3 Faktoren unterschiedlich starke Zusammenhänge mit externalisierendem Problemverhalten aufweisen. Im Kontext von externalisierendem Problemverhalten mit Freund:innen lassen sich die unterschiedlichen Zusammenhänge möglicherweise damit erklären, dass die Ausprägung von CU-Traits nicht nur einen Effekt auf die Wahrscheinlichkeit des externalisierenden Problemverhaltens hat, sondern ebenfalls auf die „Wirksamkeit“ der Gruppenprozesse, die externalisierendes Problemverhalten begünstigen. So könnte eine gleichgültige Haltung (*Uncaring*) dazu führen, dass die Reaktionen des Freundeskreises (z. B. Belohnung von externalisierendem Problemverhalten durch unterstützende Gesten) wenig(er) relevant sind und dadurch nicht dazu führen, dass vermehrt externalisierendes Problemverhalten gezeigt wird.

Außerdem deuten die vorliegenden Ergebnisse darauf hin, dass die Ausprägung von CU-Traits bei externalisierendem Problemverhalten mit Freund:innen nicht so bedeutsam ist wie bei externalisierendem Problemverhalten, das allein begangen wird – hier zeigen sich konsistent stärkere Zusammenhänge mit den Faktoren *Callousness *und *Uncaring *[16]. Der Faktor *Unemotional* scheint in beiden Fällen – ob mit Freund:innen oder allein [9, 10] – nicht konsistent mit externalisierendem Problemverhalten assoziiert zu sein. Diese inkonsistenten Befunde könnten auch darin begründet liegen, dass die Subskala *Unemotional* in der vorliegenden Form Schwächen hinsichtlich ihrer externen Validität aufweist [16]. In unterschiedlichen Studien zeigt sich, dass sie nur schwach (${\overline{r}}$ = 0,10) mit externalisierendem Problemverhalten, wie Aggression, Delinquenz und Hyperaktivität, assoziiert ist und nur schwach mit Psychopathie zusammenhängt. Eine mögliche Erklärung dafür ist, dass mit den Items der Subskala *Unemotional *primär abgeflachter Emotionsausdruck erfasst wird, der unter anderem eher mit Schüchternheit und Anhedonie als mit Psychopathie assoziiert ist [16]. Nur eines der insgesamt 5 Items bezieht sich auf Beeinträchtigungen im Emotionsempfinden beziehungsweise internale emotionale Reaktivität, bei denen die Beziehung zu CU-Traits ebenfalls noch nicht final geklärt zu sein scheint [29]. Eine Zu- bzw. Abnahme oder keine Veränderung im Emotionsempfinden oder in internaler emotionaler Reaktivität scheint abhängig von der erfassten Emotion zu sein. So weisen männliche Jugendliche mit Verhaltensproblemen und hohen Ausprägungen von CU-Traits zwar niedrigere Werte in empathischer Traurigkeit und Freude als eine unauffällige Kontrollgruppe auf, es zeigen sich jedoch keine Unterschiede in empathischer Wut [30]. Ob die Subskala *Unemotional* insgesamt zu kurz ist, um ein komplexes Konstrukt wie Emotionalität zu erfassen, und ob Items hinzugefügt werden sollten (z. B. zur Erfassung von Furchtlosigkeit), wird diskutiert. In jedem Fall kann diese methodische Schwäche eine Erklärung für den nicht signifikanten Zusammenhang mit externalisierendem Problemverhalten mit Freund:innen liefern.

Darüber hinaus ist es möglich, dass die Ergebnisse dadurch beeinflusst sind, dass die Analysen auf Subskalenebene durchgeführt wurden. So sprechen die Ergebnisse einer Metaanalyse [31] dafür, dass die Varianz der Subskalen durch den Gesamtscore beeinflusst wird. Zusätzlich berichten Kemp et al. [32], dass ein Modell mit einem übergeordneten Gesamtscore und 4 untergeordneten Faktoren (*Limited Concern, Lack of Remorse, Callous-lack of Empathy* und *Restricted Affect*) die beste Modellgüte aufweist. Die inkonsistenten Ergebnisse zeigen die Relevanz von weiterer Forschung zur Faktorstruktur des Inventory of Callous-Unemotional Traits (ICU) auf und die Ergebnisse der vorliegenden Studie sollten vor diesem Hintergrund interpretiert werden.

Bei der Interpretation der Ergebnisse muss ebenfalls berücksichtigt werden, dass nicht betrachtet wurde, ob es sich bei den Teilnehmenden der Studie um delinquente Jugendliche beziehungsweise Jugendliche mit (diagnostizierten) Verhaltensproblemen handelt. In einigen der Studien zu CU-Traits wurden explizit nur delinquente Jugendliche oder Kinder/Jugendliche betrachtet, bei denen (diagnostizierte) Verhaltensprobleme vorlagen und die zusätzlich hohe beziehungsweise niedrige Ausprägungen von CU-Traits aufwiesen [10, 17, 29, 32–35]. Über diese Gruppen können auf Grundlage der hier gefundenen Ergebnisse keine Aussagen getroffen werden. Zudem ist die Stichprobe hinsichtlich ihrer soziodemografischen Merkmale (z. B. Schulform, Geburtsland und Muttersprache) weniger heterogen. So berichtete etwas mehr als die Hälfte der Jugendlichen, dass sie ein Gymnasium besuchen, und ein Großteil gab an, in Deutschland geboren zu sein und zu Hause hauptsächlich Deutsch zu sprechen. Dies schmälert die Generalisierbarkeit der Ergebnisse. In weiterführender Forschung sollten die Ergebnisse in einer heterogeneren Stichprobe und besonders auch für delinquente Jugendliche und Jugendliche mit diagnostizierten Verhaltensproblemen in Kombination mit niedrigen/hohen Ausprägungen von CU-Traits validiert werden.

Darüber hinaus ist anzumerken, dass es sich bei der Datenerhebung um eine Selbstbeurteilung handelte, bei der sozial erwünschte Antworten nicht vollständig ausgeschlossen werden konnten, und auch das Klassensetting kann zu Verzerrungen der Ergebnisse geführt haben. Eine Skala zur Erfassung der sozialen Erwünschtheit [36] wurde miterhoben, zeigte jedoch keine Zusammenhänge mit den Studienvariablen. Nichtsdestotrotz ist es für angeschlossene Forschung sinnvoll, zum Beispiel die Fremdbeurteilungsversionen der ICU mitzuerheben. Ebenfalls wichtig zu beachten ist, dass eine etablierte Skala zur Erfassung von externalisierendem Problemverhalten mit Freund:innen modifiziert wurde. Es zeigt sich zwar eine exzellente interne Konsistenz, die Skala wurde vorher jedoch noch nicht verwendet und ist nicht validiert.

### Praktische Implikationen

Für die Prävention von externalisierendem Problemverhalten, im Speziellen von regelverletzendem und dissozialem Verhalten mit Freund:innen, deuten die Ergebnisse darauf hin, dass auf individueller Ebene ein Mangel an Empathie und Reue ein möglicher Ansatzpunkt ist. Im Folgenden werden exemplarisch 2 soziale Kompetenztrainings beschrieben, die im Jugendalter anwendbar sind, jedoch für unterschiedliche Zielgruppen konzipiert wurden.

Im „WiSK-Programm“ ist die Förderung von Empathie als konkretes Ziel benannt und es dient der Förderung von sozialen und interkulturellen Kompetenzen sowie der Gewalt‑/Mobbingprävention [37]. Es basiert auf einem Mehrebenenansatz, der die Schule, die Klasse und einzelne Schüler:innen einbezieht. Auf Klassen- und Individualebene soll Empathie explizit gefördert werden. Die Maßnahmen auf Individualebene setzen intervenierend an und im Fokus stehen Gespräche. In den Gesprächen mit Täter:innen liegt neben der Förderung von Empathie der Fokus darauf, ihnen zu vermitteln, dass Gewalt nicht toleriert wird und dass sie Verantwortung für das eigene Handeln übernehmen müssen. Studien zur Wirksamkeit des WiSK-Programms zeigten, dass Teilnehmende seltener Opfer von Aggression waren als Schüler:innen der Kontrollgruppe [38] und Cybermobbing sowie Cyberviktimisierung reduziert wurden [39, 40], wobei die Implementierungsqualität einen Einfluss auf die Effektivität des Programms zu haben schien [41].

Ein Interventionsprogramm, das explizit für delinquente Jugendliche konzipiert wurde und die Fähigkeit zur Perspektivübernahme als wichtige Voraussetzung für Empathie fördern soll, ist das Trainingsprogramm zur Aggressionsverminderung (TAV; [42]). Das theoretisch fundierte [43] Ziel des Programms ist es, Aggressionen dadurch zu reduzieren, dass die Einstellungen zu aggressivem Verhalten verändert werden und ein prosoziales Verhaltensrepertoire aufgebaut wird. Das TAV ist als Gruppenprogramm konzipiert und basiert auf sozialen Problemen, die von den Jugendlichen gelöst und reflektiert werden sollen, wobei die Perspektivübernahme eine bedeutsame Rolle spielt. Anschließend werden die Problemlösestrategien in Rollenspielen ausprobiert und trainiert. Das TAV führt zur Veränderung der Einstellungen gegenüber Aggression und aggressiven Verhaltensweisen sowie vermehrtem Mitgefühl [42].

### Fazit und Ausblick

Die Ergebnisse der vorliegenden Studien zeigen zum einen die Relevanz davon auf, die 3 Facetten der Callous-Unemotional Traits (CU-Traits) getrennt voneinander zu betrachten, und zum anderen, dass bei der Untersuchung von externalisierendem Problemverhalten zu berücksichtigen ist, ob es sich um Verhalten handelt, dass allein oder gemeinsam mit beziehungsweise in Anwesenheit von Freund:innen gezeigt wird.

Aufgrund der geringen Varianzaufklärung von 6 % in der vorliegenden Studie ist allerdings davon auszugehen, dass über die CU-Traits hinaus weitere Faktoren bedeutsam sind. Neben den methodischen Erklärungen lässt sich der geringe Anteil von erklärter Varianz durch die CU-Traits bzw. den Faktor *Callousness* möglicherweise damit erklären, dass individuelle Persönlichkeitsmerkmale – wie die CU-Traits – durch die Anwesenheit der Freund:innen weniger relevant sind. Eventuell werden Empathie, Mitgefühl und Schuldgefühle im Gruppenkontext, zum Beispiel aufgrund der Verantwortungsdiffusion, weniger/nicht aktiviert und das unabhängig von Ausprägungen der CU-Traits. Dies sollte in weiteren Forschungsarbeiten überprüft werden, in denen dann explizit nach externalisierendem Problemverhalten mit Freund:innen gefragt wird. Dabei sollte darauf geachtet werden, welche Quelle(n) zur Messung des externalisierenden Problemverhaltens herangezogen werden. So zeigt sich, dass externalisierendes Problemverhalten überschätzt wird, wenn es fremdberichtet wird (z. B. wenn Jugendliche es bei ihren Freund:innen einschätzen sollen). Dies scheint mit dem eigenen externalisierenden Problemverhalten der einschätzenden Jugendlichen zusammenzuhängen, das bedeutet, dass bei eigenem häufigen externalisierenden Problemverhalten das der Freund:innen auch als häufig eingeschätzt wird [44]. Eventuell trifft dies auch auf die Messung von externalisierendem Problemverhalten mit Freund:innen zu, daher sollte möglichst mehr als eine Quelle befragt werden, zum Beispiel durch die Erhebung von Netzwerkdaten innerhalb von Freundeskreisen. Darüber hinaus wurde in der vorliegenden Studie nicht zwischen den Rollen differenziert, die Jugendliche im Rahmen von externalisierendem Problemverhalten mit Freund:innen einnehmen können. Sie können zum Beispiel gemeinsam mit ihren Freund:innen aktiv externalisierendes Problemverhalten zeigen („aktive Rolle“) oder selbst nicht aktiv handeln und „nur dabei sein“ (Bystander). Forschung zu Mobbing zeigt, dass moralische Rechtfertigungen mit direktem Mobbing, nicht aber mit proaggressivem Bystander-Verhalten assoziiert sind [45], eventuell zeigen sich diese Unterschiede auch für den Zusammenhang mit CU-Traits. Insgesamt sollten die Ergebnisse vor dem Hintergrund betrachtet werden, dass es sich um die erste Studie handelt, in der der Zusammenhang von CU-Traits und externalisierendem Problemverhalten mit Freund:innen betrachtet wurde. Sie machen jedoch deutlich, wie relevant weiterführende Forschung mit diesem Fokus ist.
